# Ethylenediaminetetraacetic acid as capping ligands for highly water-dispersible iron oxide particles

**DOI:** 10.1186/1556-276X-9-27

**Published:** 2014-01-14

**Authors:** Yunfeng Yi, Ying Zhang, Yixiao Wang, Lihua Shen, Mengmeng Jia, Yu Huang, Zhenqing Hou, Guohong Zhuang

**Affiliations:** 1 The Affiliated Southeast Hospital of Xiamen University, Zhangzhou 363000, China; 2 Department of Radiology, Taishan Medical University, Taishan, Shandong 271016, China; 3 Research Center of Biomedical Engineering, Department of Biomaterials, College of Materials, Xiamen University, Xiamen 361005, China; 4 Organ Transplantation Institute, Medical College, Xiamen University, Xiamen 361005, China; 5 Anti-Cancer Research Center, Medical College, Xiamen University, Xiamen 361005, China

**Keywords:** Magnetite, Magnetic properties, Nanocrystalline materials

## Abstract

Monodispersed magnetite (Fe_3_O_4_) particles were synthesized using a high-temperature hydrolysis reaction with the assistance of ethylenediaminetetraacetic acid (EDTA) as capping ligands. These particles were composed of small primary nanocrystals and their sizes could be tuned from about 400 to about 800 nm by simply changing the EDTA or precursor concentration. Surface-tethered EDTA made the particles highly water-dispersible. The as-prepared magnetite particles also showed superparamagnetic behavior at room temperature, and their magnetic properties were size dependent. In addition, the particles had a strong response to external magnetic field due to their high magnetization saturation values. These properties were very important for some potential biomedical applications, such as magnetic separation and magnetic-targeted substrate delivery.

## Background

Over the past decade, magnetic nanocrystals (e.g., Fe_3_O_4_, γ-Fe_2_O_3_) have attracted much attention due to their unique magnetic properties and important applications such as targeted drug delivery [1,2], biomolecular separations [3,4], treatment of hyperthermia in cancer [5,6], and as contrast agents in magnetic resonance imaging (MRI) [7,8]. Up to now, many methods have been developed to prepare Fe_3_O_4_ nanocrystals with small sizes on the nanometer scale, which include hydrothermal synthesis [9,10], chemical coprecipitation [11-13], and thermal decomposition and/or reduction [14,15]. Besides these nanosized particles, the secondary structural superparamagnetic Fe_3_O_4_ particles have also attracted increasing attention due to their practical applications in magnetic separation and magnetic-targeted substrate delivery [16,17]. Generally, these secondary structural Fe_3_O_4_ particles consist of small Fe_3_O_4_ nanocrystals. As-prepared Fe_3_O_4_ particles are stable in solution and reveal rapid magnetic response to the externally applied magnetic field. Over the past decade, these secondary structural Fe_3_O_4_ particles are prepared by a common two-step process, including cooperative assembly [18], microemulsion templating [19], and spontaneous assembly [20]. Compared to the two-step process of assembling the pre-synthesized Fe_3_O_4_ nanocrystals into uniform secondary structures, the direct one-step growth route to synthesize the secondary structural Fe_3_O_4_ particles seems to be a simpler way, which is also economical for large-scale production.

Herein we reported a general approach for the fabrication of monodispersed, highly water-dispersible, and superparamagnetic Fe_3_O_4_ particles by a one-step hydrothermal procedure using an ethylenediaminetetraacetic acid (EDTA)-assisted route. Biocompatible EDTA was chosen because it can act as a crystal grain growth inhibitor for the synthesis of variously sized Fe_3_O_4_ particles, and the carboxylate groups of EDTA have a strong coordination affinity to the iron cations on the Fe_3_O_4_ surface, which might favor the attachment of hydrophilic groups on the surface of the Fe_3_O_4_ particles. Herein, the Fe_3_O_4_ particles synthesized with the assistance of EDTA were also intrinsically stabilized with a layer of hydrophilic ligand *in situ*, which was essential for their long-term stability in aqueous media without any surface modification.

## Methods

### Synthesis of Fe_3_O_4_ particles

In a typical synthesis of 725 nm Fe_3_O_4_ particles, 1.3 g of anhydrous FeCl_3_ was first vigorously mixed with 40 mL of ethylene glycol (EG) to form a clear solution. Then, 0.47 g of EDTA was added and the mixture was heated at 110°C, followed by dissolving of anhydrous sodium acetate (NaOAc) (2.4 g), Then the mixture was transferred into a 100-mL Teflon-lined stainless-steel autoclave and sealed in air. The autoclave was kept at 200°C for 10 h. The black products were collected by a magnet and washed with ethanol three times, and the products were dried at 60°C for further use.

### Characterizations

The x-ray diffraction (XRD) patterns were collected between 20° and 80° (2*θ*) on an x-ray diffraction system (X’Pert Pro, PANalytical Co., Almelo, The Netherlands) with a graphite monochromator and Cu Kα radiation (*λ* = 0.15406 nm). Transmission electron microscope (TEM) images and selected area electron diffraction (SAED) patterns were obtained (JEOL JEM-2100; JEOL, Tokyo, Japan) operated at an accelerating voltage of 200 kV. The samples for TEM and high-resolution transmission electron microscope (HR-TEM) analyses were prepared by spreading a drop of as-prepared magnetite nanoparticle-diluted dispersion on copper grids coated with a carbon film followed by evaporation under ambient conditions. Atom force microscope (AFM) characterization was carried out using Scan Asyst-Air (Bruker Multimode 8, Bruker Corporation, Billerica, MA, USA). Measurements were carried out in air, and imaging was performed in tapping mode. The height, amplitude, and phase images were recorded. The scanning electron microscopy (SEM) images were obtained using LEO 1530 microscope (LEO, Munich, Germany).

## Results and discussion

**Figure 1:**
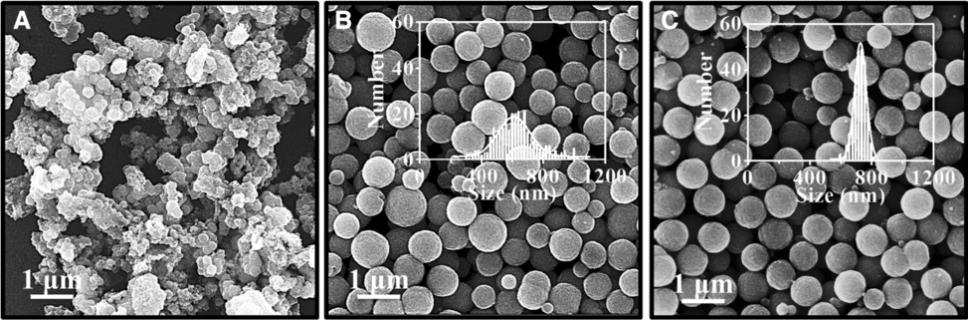
**TEM images of Fe**_**3**_**O**_**4 **_**particles synthesized with different FeCl**_**3 **_**concentrations. (A)** 0.05. **(B)** 0.10. **(C)** 0.20 mol L^−1^. Inset is the corresponding particle size distribution.

The morphology of the as-prepared Fe_3_O_4_ particles was characterized by SEM (Figure 1). As shown in Figure 1A, when FeCl_3_ concentration is low (0.05 mol L^−1^), the products are nonuniform, consisting of spherical nanocrystal clusters and small nanocrystal aggregations. However, when the FeCl_3_ concentration is in the range of 0.10 to 0.20 mol L^−1^, all of Fe_3_O_4_ particles have a nearly spherical shape (Figure 1B,C). The diameters of the particles slightly increase from 622 ± 145 nm to 717 ± 43 nm, but their sizes become more uniform with the increase of FeCl_3_ concentration, indicating that higher FeCl_3_ concentrations could lead to a larger and more uniform particle size.

**Figure 2:**
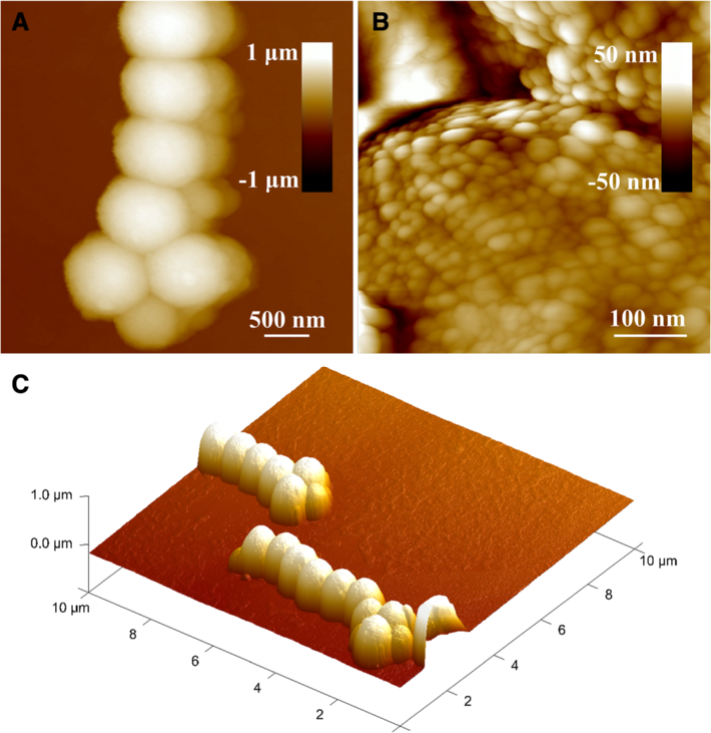
**Surface morphology of the as-obtained Fe3O4 particles. (A)** AFM image of Fe_3_O_4_ particles. **(B)** The enlarged AFM image of the isolated particles. **(C)** 3D image reconstruction of Fe_3_O_4_ particles.

The surface morphology of the as-obtained Fe_3_O_4_ particles is further shown in Figure 2. The 2D and 3D AFM images of Fe_3_O_4_ particles prepared from 0.20 mol L^−1^ of FeCl_3_ appear a nearly uniform size of about 725 nm and spherical shape, which is in good agreement to the SEM results (Figure 1C). Furthermore, a high-resolution AFM image of an isolated Fe_3_O_4_ particle (Figure 2B) also indicates that the as-prepared Fe_3_O_4_ particles are composed of small nanocrystals with the size of about 7 to 15 nm.

**Figure 3:**
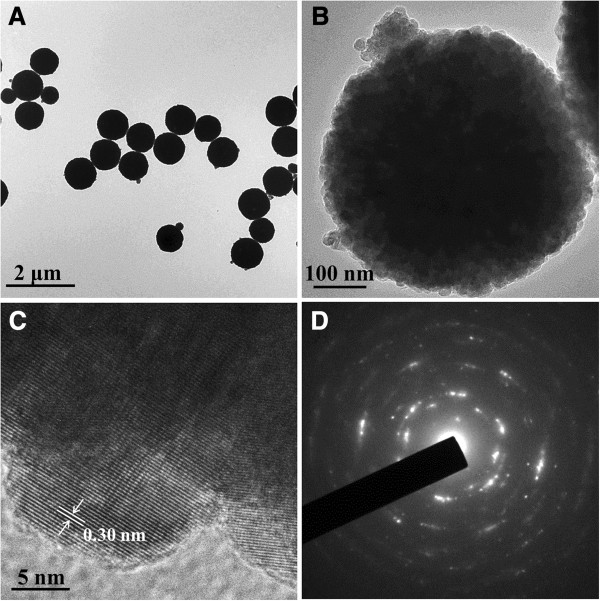
**Uniform sizes and morphology of the as-prepared Fe**_**3**_**O**_**4 **_**particles.** TEM images **(A, B)** and HR-TEM image **(C)** of the as-prepared Fe_3_O_4_ particles. SAED pattern of the particle in B **(D)**.

TEM image of the as-prepared Fe_3_O_4_ particles (Figure 3A) further demonstrates their uniform sizes and morphology. The secondary structure of Fe_3_O_4_ particles also could be observed more clearly in Figure 3B for the isolated cluster, indicating that the obtained Fe_3_O_4_ particles are compact clusters. The HR-TEM image recorded at the edge of the Fe_3_O_4_ particles is shown in Figure 3C. Measuring the distance between two adjacent planes in a specific direction gives a value of 0.30 nm, corresponding to the lattice spacing of (220) planes of cubic magnetite [21,22]. The SAED pattern (Figure 3D) shows polycrystalline-like diffraction, suggesting that the as-prepared Fe_3_O_4_ particles consist of magnetite nanocrystals.

**Figure 4:**
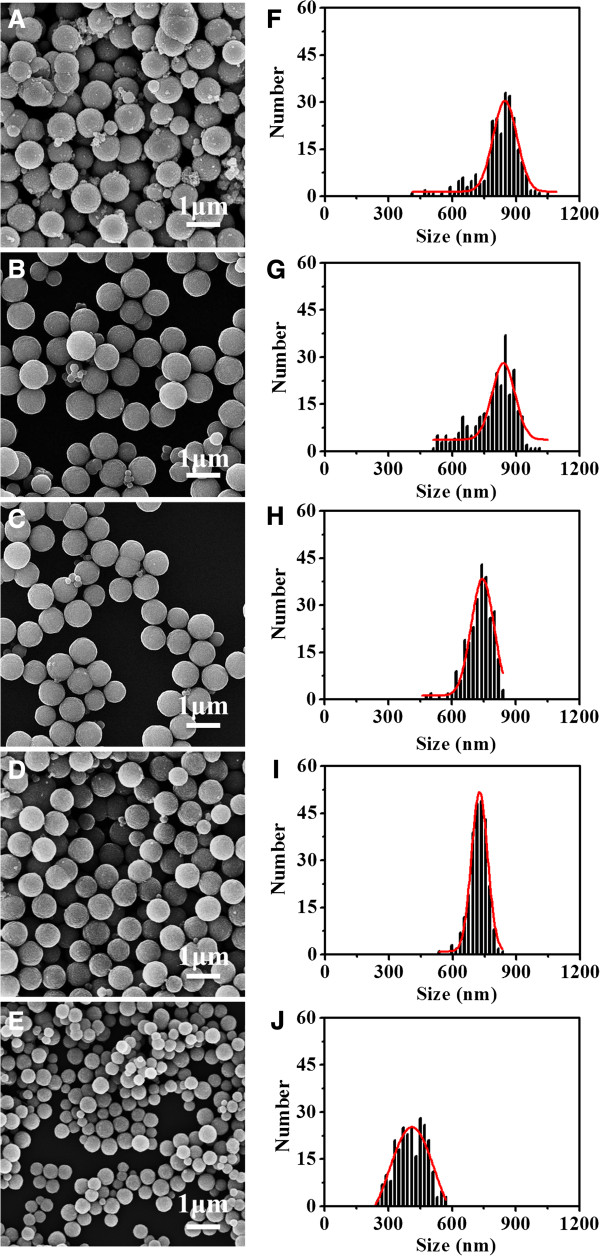
**TEM images and XRD patterns of Fe**_**3**_**O**_**4 **_**particles. (A-E)** TEM images and **(F-J)** XRD patterns of Fe_3_O_4_ particles synthesized with different EDTA concentrations: 0, 10, 20, 40, and 80 mol L^−1^, respectively.

The effects of EDTA concentration on the particle sizes and grain sizes of Fe_3_O_4_ particles are further investigated. Without addition of EDTA, the resultant products have a heterogeneous size distribution and their shapes are nonuniform (Figure 4A,F). When the initial EDTA concentration is increased from 10 to 40 mmol L^−1^, the sizes of Fe_3_O_4_ particles decrease slightly from 794 ± 103 nm to 717 ± 43 nm (Figure 4B,C,D and 4G,H,I) and their size distribution becomes more uniform. However, when the EDTA concentration further increases to 80 mmol L^−1^, their sizes decrease significantly to 409 ± 70 nm while their size distribution becomes heterogeneous again (Figure 4E,J), indicating that higher EDTA concentration favors the formation of Fe_3_O_4_ particles with larger size; their size distribution, however, is EDTA concentration dependent.

**Figure 5:**
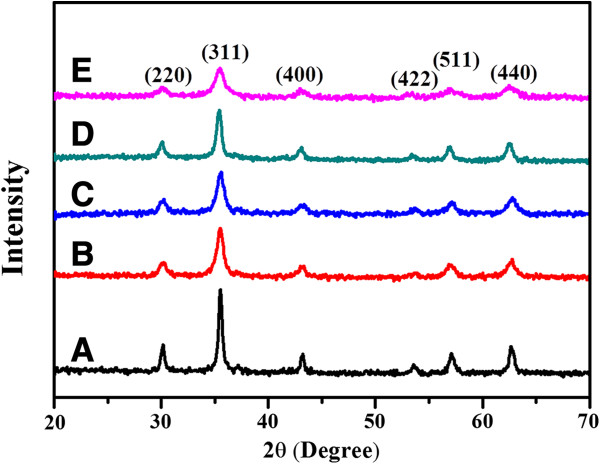
**XRD patterns of Fe**_**3**_**O**_**4 **_**particles synthesized with different EDTA concentrations.** (A) 0, (B) 10, (C) 20, (D) 40, and (E) 80 mol L^−1^, respectively.

To confirm the effects of EDTA concentration on the grain sizes and the corresponding crystalline structures and phase composition of the as-prepared Fe_3_O_4_ particles, the samples obtained with different EDTA concentrations are characterized by XRD. As shown in Figure 5, all the diffraction peaks are indexed to the spinel structure, known for the Fe_3_O_4_ crystal (JCPDS no. 00-003-0863) and no other peaks are detected, indicating that the products are pure phase Fe_3_O_4_. In addition, as the EDTA concentration increases, the broadening in the diffraction peaks becomes more pronounced. The grain sizes of the Fe_3_O_4_ particles calculated from the breadth of the (311) reflection using Debye-Scherrer’s formula [23,24] decrease dramatically from 14.8 to 7.6 nm when the initial EDTA concentration increases from 0 to 80 mmol L^−1^. It is thus concluded that EDTA could act as a stabilizer, which might significantly suppress the grain growth of the as-synthesized Fe_3_O_4_ particles.

**Figure 6:**
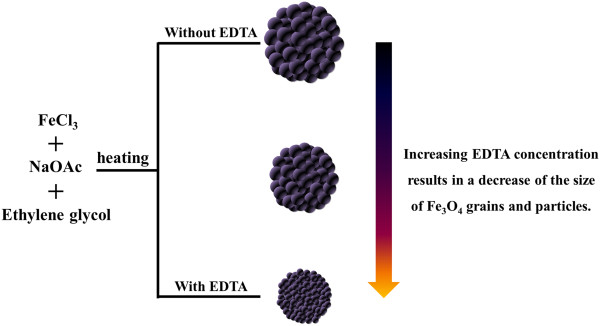
**Schematic representation of the formation of Fe**_**3**_**O**_**4 **_**particles with tunable grain size and particle size.**

**Figure 7:**
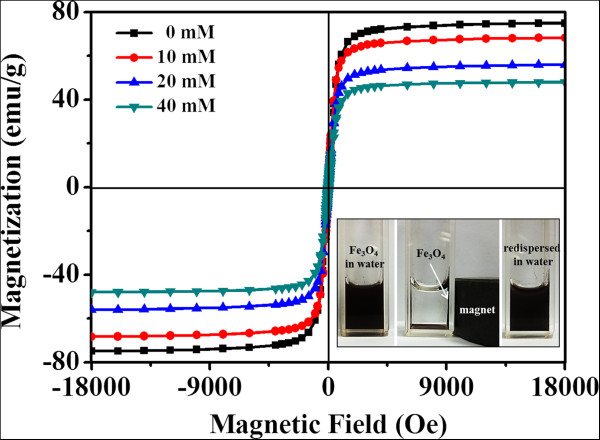
**Hysteresis loops of the Fe**_**3**_**O**_**4 **_**particles obtained with EDTA concentrations.** Inset was the photographs of an aqueous solution of Fe_3_O_4_ particles without magnetic field and with the externally applied magnetic field.

As a consequence, a probable mechanism which leads to the resulting Fe_3_O_4_ particles with tunable grain size and particle size is proposed as follows (Figure 6). When EDTA is introduced to the FeCl_3_/EG solution, a significant amount of Fe-EDTA complex is formed. NaOAc is then added and utilized as an alkali source. In the presence of EG and EDTA, Fe_3_O_4_ crystallites are formed first under alkaline condition, followed by further growth into Fe_3_O_4_ nanoparticles as the prolonging of reaction time in this system. The primary Fe_3_O_4_ nanoparticles then gradually aggregate into large particles to minimize the surface energy. In addition, because of the strong coordination between Fe(III) ions and carboxylate on the surface of particles [9,14,25], the as-prepared Fe_3_O_4_ particles also possess a coating of carboxylate and could be easily dispersed in water (inset in Figure 7). When a magnet is applied, the particles could be completely separated from the solution within seconds. Once the magnet is withdrawn, the particles could be redispersed into the water immediately by slight shaking. Furthermore, by increasing the amount of EDTA, more carboxylate groups could bind to the surface of Fe_3_O_4_ particles through the strong coordinating ligand. This results in a decrease of the size of Fe_3_O_4_ grains and particles. Magnetic properties (*M*-*H* curves) of Fe_3_O_4_ particles synthesized with EDTA over the concentration range of 0 to 40 mmol L^−1^ are shown in Figure 7. It is obvious that all the Fe_3_O_4_ particles have no remanence or coercivity at 300 K and their magnetic properties are strongly dependent on the sizes of Fe_3_O_4_ particles prepared. When the initial EDTA concentration is increased from 10 to 40 mmol L^−1^, the sizes of Fe_3_O_4_ particles slightly decrease from 794 ± 103 nm to 717 ± 43 nm. Their magnetization saturation (Ms) values simultaneously suffer a corresponding decrease from 74.9 to 48.0 emu g^−1^. This result also suggests that lower EDTA concentration favors the formation of Fe_3_O_4_ particles with better crystallinity, which is in good agreement to the XRD results.

## Conclusions

In summary, a modified solvothermal approach was used to synthesize monodispersed Fe_3_O_4_ particles with the assistance of EDTA, which are composed of numerous primary Fe_3_O_4_ nanocrystals with sizes of 7 to 15 nm. Their sizes could be easily tuned over a wide range of 400 to 800 nm by simply varying the concentration of FeCl_3_ or EDTA. More importantly, owing to the presence of the carboxylate groups attached on the surface, the Fe_3_O_4_ particles have excellent water dispersibility and dispersing stability. In addition, the growth mechanism of the secondary structural Fe_3_O_4_ particles is discussed. The magnetite particles are also superparamagnetic at room temperature and have a high magnetization, which enhance their response to external magnetic field and therefore should greatly facilitate the manipulation of the particles in practical uses.

## Competing interests

The authors declare that they have no competing interests.

## Authors’ contributions

YY, YZ, and MJ performed the experiments. YW, LS, and YH were involved in experimental planning and analysis of the results. ZH and GZ designed and planned the experiment and LS also drafted the manuscript. All authors read and approved the final manuscript.
